# Transperineal laser ablation (TPLA) of the prostate for benign prostatic obstruction: the first 100 patients cohort of a prospective, single-center study

**DOI:** 10.1007/s00345-024-05077-z

**Published:** 2024-07-10

**Authors:** Mattia Lo Re, Paolo Polverino, Anna Rivetti, Alessio Pecoraro, Marco Saladino, Marta Pezzoli, Giampaolo Siena, Cosimo De Nunzio, Vincenzo Li Marzi, Mauro Gacci, Sergio Serni, Riccardo Campi, Francesco Sessa

**Affiliations:** 1 Unit of Urological Robotic Surgery and Renal Transplantation, University of Florence, Careggi Hospital, Florence, 50100 Italy; 2 Unit of Oncologic Minimally Invasive Urology and Andrology, University of Florence, Careggi Hospital, Florence, 50100 Italy; 3 Department of Urology, Sant’Andrea Hospital, Sapienza University, Rome, Italy; 4 Department of Experimental and Clinical Medicine, University of Florence, Florence, Italy

**Keywords:** Transperineal laser ablation of the prostate, Benign prostatic hyperplasia, Lower urinary tract symptoms, Ultra-minimally invasive surgical techniques, Ejaculation sparing

## Abstract

**Purpose:**

Transperineal laser ablation (TPLA) is a new minimally-invasive surgical treatment for patients with benign prostatic obstruction (BPO). We report the perioperative and mid-term functional results of the first 100 consecutively patients undergoing TPLA at our institution.

**Methods:**

Clinical data from consecutive patients undergoing TPLA at our institution from April 2021 to July 2023 were prospectively collected. Primary endpoints were the postoperative changes in IPSS, QoL and MSHQ 3-item questionnaires and in Qmax and post-void residual volume (PVR).

**Results:**

Overall, 100 consecutive patients underwent the procedure. Median age and prostate volume were 66 (IQR 60–75) years and 50 (IQR 40–70) ml, respectively. In the cohort, 14 (14%) patients had an indwelling catheter and 81 (81%) were under oral BPO therapy at the time of TPLA. Baseline median Qmax (ml/s) and PVR (ml) were 9.1 (IQR 6.9–12) and 90 (IQR 50–150), respectively, while median IPSS and QoL were 18 (IQR 15–23) and 4 (IQR 3–4). At all the follow-up timepoints, the evaluated outcomes on both symptoms and functional parameters showed a statistically significant improvement (*p* < 0.001). Antegrade ejaculation was preserved in all sexually active patients. No postoperative Clavien-Dindo > 2 complications were recorded.

**Conclusions:**

TPLA represents a safe option for selected well-informed patients swith LUTS due to BPO. Our prospective study confirms the feasibility and favorable perioperative and functional outcomes in a real-world cohort with heterogenous prostate volumes and patient characteristics.

**Supplementary Information:**

The online version contains supplementary material available at 10.1007/s00345-024-05077-z.

## Introduction

Benign Prostatic Hyperplasia (BPH) is a relevant medical condition among aging males, due to enlargement of the prostate gland, leading to lower urinary tract symptoms (LUTS) [1]. With the increase in life expectancy and an aging population, BPH has become a significant healthcare concern, impacting the quality of life and healthcare resources [2].

The main guidelines suggest that the primary approach to the pathology involves lifestyle changes, followed by the use of pharmacological therapy, and eventually, surgical intervention [3]. As surgical treatment, the European Association of Urologist (EAU) Guideline considers Transurethral Resection of the Prostate (TURP) as the gold standard for prostates up to 80 ml in size, while prostate enucleation is the best option for patients with larger prostates [4]. However, these procedures are associated with a non-negligible risk of perioperative and long-term complications and side effects such as retrograde ejaculation, infection, urinary sepsis, hematuria, urethral strictures and urinary incontinence [5–10].

In recent years, there has been a growing interest from both patients and clinicians for minimally invasive and ultra-minimally invasive surgical techniques (MISTs and uMISTs), aiming to minimize side effects in favor of an approach that ensures favorable efficacy outcomes [11, 12]. In this context, the ultrasound-guided SoracteLite™ transperineal laser ablation (TPLA) represents a new option for minimally-invasive surgical treatment of patients with BPH with potential advantages for patients and healthcare systems [13, 14]. To evaluate the potential and the effect of this technique, we developed a prospective descriptive study and herein, we report the perioperative and mid-term functional results of the first monocentric cohort of 100 patients treated with TPLA in our institution.

## Materials and methods

After Institutional Review Board approval and obtained patients’ written informed consent after comprehensive shared decision-making regarding all available alternative therapeutic options, data from all consecutive patients undergoing TPLA at our institution between April 2021 and July 2023 were prospectively collected in a dedicated database.

Inclusion criteria were (1) age ≥ 45 years; (2) moderate to severe LUTS due to BPO with an International Prostate Symptom Score, (IPSS) score ≥ 8; (3) prostate volume ≥ 30 mL and ≤ 100 ml measured via transabdominal Ultrasound or MRI; (4) ineffectiveness of medical therapies due to lack of efficacy, intolerance, poor compliance or strong desire to preserve antegrade ejaculation or very high risk for standard surgery due to comorbidities.

On the other hand, the main exclusion criteria were (1) clinical suspicion of prostate cancer or prostate cancer history, (2) neurogenic bladder disfunctions, (3) urethral strictures, (4) bladder stones, (5) large median lobe (Intravesical Prostatic Protrusion over 1,5 cm), (6) previous prostatic surgery.

Patients with an indwelling catheter were considered eligible for TPLA after performing an invasive urodynamic assessment that excluded severe detrusor hypo-contractility.

All patients underwent a standardized preoperative diagnostic work-up including digital exploration, serum PSA and, in case of suspected prostate cancer, multiparametric magnetic resonance imaging of the prostate (mpMRI). Flexible cystoscopy was performed at the physician’s discretion in case of unclear indications for MISTs (e.g. suspected significant third lobe, suspected bladder cancer, etc.).

Patient age, body mass index (BMI), Charlson Comorbidity Index (CCI), anticoagulant/antiplatelet medication, BPH medical history, were recorded.

At baseline, 3, 6, 12 and last follow-up validated questionnaires results including international index of erectile function (IIEF-5), Quality of life (QoL), International Prostate Symptom Score (IPSS), Male Sexual Health Questionnaire-Ejaculatory Dysfunction (MSHQ-EjD) 3-items were collected for all patients. Moreover, data about non-invasive urodynamic analyses to assess maximum flow at uroflowmetry (Qmax) and post-void residual (PVR) were recorded.

TPLA was performed in an outpatient setting using local anaesthesia (20mL Lidocaine 2%) and low-dose oral benzodiazepine administration according to patients’ preference, using EchoLaser™ multisource diode laser generator for the ablation. After the positioning of a transurethral catheter and local disinfection, one or two 21G needle were introduced transperineally and located in the middle of each lobe, under ultrasound guidance, with its orientation parallel to the longitudinal axis of the gland.

Before starting the treatment security distances from the urethra (8 mm, thus preventing possible damages resulting in hematuria, storage LUTS and lumen stenosis) and from the bladder neck (around 15 mm, critical to avoid ejaculatory dysfunction) were checked. The procedure was then planned thanks to the Echolaser Smart Interface (ESI), a dedicated device with a planning software connected with the video output of the US system, for real-time user assistance in performing the procedure. The 300 micrometers disposable optical fibers were then introduced. After the insertion of the applicators, a check of their safe position was performed with ESI. The starting power energy was 5 W, reduced in about 2 min to 3,5 W. A more accurate description of the technique could be found in previous experience [15].

Primary endpoints were the IPSS, QoL, Qmax, PVR and MSHQ – EjD 3-items at 3, 6 and 12 months. Complications were recorded and classified according to the Clavien-Dindo scale. Discontinuation and reintroduction of BPH medical therapy was recorded.

Treatment failure after the procedure was defined as the need to shift to other invasive surgical treatment for BPH due to relapse of symptoms or non-negligible worsening of functional outcomes.

Statistical analyses were performed using IBM SPSS statistics 27 (IBM SPSS, IBM Corp., Armonk, NY, USA). Values for quantitative variables are expressed as median and interquartile range (IQR). Comparisons between pair of values (baseline - each time point) were performed using a Wilcoxon signed rank test, with a p-value < 0.05 deemed to be statistically significant.

## Results

A total of 100 patients, with a median age of 66 (IQR 60–75) years with symptomatic BPH underwent the procedure. Preoperative median prostate volume was 50 (IQR 40–70) ml and 14 (14%) patients had urinary catheter before the procedure. Eighty-one (81%) patients were taking medical therapy for their LUTS at the time of the surgery (alpha-blockers, 5-ARI or combined therapy), while 19 (19%) were not assuming any drugs. Baseline median Qmax and PVR were 9.1 mL/s (IQR 6.9–12) and 90 mL (IQR 50–150), respectively; baseline median IPSS and IPSS-QoL were 18 (IQR 15–23) and 4 (IQR 3–4); baseline median MSHQ 3-items was 6 (IQR 2–11) (Table 1). Ninety-nine patients were discharged within daily hospital stay; one patient - treated in the afternoon - required overnight hospitalization for pelvic pain and was discharged on postoperative day 1. Median catheterization time was 7 days (IQR 7–7). Median follow up was 12 months (IQR 6–18). No intraoperative complications were recorded; 2 (2%) patients experienced urinary tract infection treated with oral antibiotics in the first 3 months after the procedure (Clavien-Dindo 2). No postoperative Clavien-Dindo 3–5 complications were recorded.

**Table 1:** Preoperative patients characteristics and intraoperative features

Preoperative characteristics	*n* = 100
Age (years); median (IQR)	66.5 (60–75)
BMI (kg/m^2^); median (IQR)	25.9 (23.5–27.6)
ASA score; <2	55 (55%)
CCI score (not age adjusted); median (IQR)	1 (0–2)
Prostate volume (mL); median (IQR)	50 (40–70)
Patients with indwelling catheter; n (%)	14 (14%)
Patients under antiplatelet/anticoagulant therapy; n (%)	24 (24%)
Patients under BPH therapy; n (%)	81 (81%)
Alpha-blockers; n (%)	60 (60%)
5 – ARI; n (%)	4 (4%)
Combined therapy; n (%)	17 (17%)
Baseline Qmax (mL/s), median (IQR)	9.1 (6.9–12)
Baseline PVR, median (IQR)	90 (50–150)
Baseline IPSS, median (IQR)	18 (15–23)
Baseline QoL, median (IQR)	4 (3–4)
Baseline MSHQ 3-item, median (IQR)	6 (2–11)
**Intraoperative features**	
Number of fibers; median (IQR)	2 (2–2)
Energy erogated; median (IQR)	2800 (2400–3100)
Catheterization time (days); median (IQR)	7 (7–7)

BMI: body mass index; IQR: interquartile range; ASA: American Society of Anesthesiologists; CCI: Charlson Comorbidity Index; 5-ARI: 5-alpha-reductase inhibitors; Qmax: maximum flow rate; PVR: post-void residual; IPSS: International Prostatic Symptoms Score; QoL: Quality of Life; MSHQ: men sexual health questionnaire

At each timepoint, the previously described outcomes on both symptoms (IPSS, QoL, MSHQ 3-items) and urodynamics parameters (Qmax, PVR) showed a statistically significant improvement (all p value < 0.001) (Table 2).

**Table 2:** Functional outcomes

	Baseline	3 months	*p*	6 months	*p*	12 months	*p*
**Median Qmax (mL/s) (IQR)**	9.1 (6.9–12)	11 (8.8–14.8)		11 (8.5–16.0)		13 (8.5–16.9)	
**Median ΔQmax (mL/s) (IQR)**		2.4 (0.1–4.4)	**< 0.001**	2.5 (0.8–5.9)	**< 0.001**	3.9 (1.6–7.3)	**< 0.001**
**Median IPSS (IQR)**	18 (15–23)	10 (6–13)		10 (5.7–14)		10 (5–16.5)	
**Median ΔIPSS (IQR)**		-9 (-13 - -5)	**< 0.001**	-9 (-13 - -4)	**< 0.001**	-9 (-16 - -3)	**< 0.001**
**Median QoL (IQR)**	4 (3–4)	2 (1–3)		2 (1–3)		2 (1–3)	
**Median ΔQoL (IQR)**		-2 (-3 - -1)	**< 0.001**	-2 (-3 - -1)	**< 0.001**	-2 (-1 - -3)	**< 0.001**
**Median MSHQ 3 item (IQR)**	6 (2–11)	10 (5–13)		11 (5–14)		9 (5–13)	
**Median ΔMSHQ 3 item (IQR)**		2 (0–4)	**< 0.001**	2 (0–5)	**< 0.001**	4 (1–5)	**< 0.001**
**Median PVR (mL) (IQR)**	90 (50–150)	45 (20–77,5)		50 (20–90)		45 (1.2–87.5)	
**Median ΔPVR (IQR)**		-45 (-82.5 - -7.5)	**< 0.001**	-50 (-92.5–0)	**< 0.001**	-60 (-103.7 - -22)	**< 0.001**

IQR: interquartile range; Qmax: maximum flow rate; PVR: post-void residual; IPSS: International Prostatic Symptoms Score; QoL: Quality of Life; MSHQ: men sexual health questionnaire

Specifically, median Qmax (ml/s) was 11 (8.8–14.8), 11 (8.5–16.0) and 13 (8.5–16.9) at 3, 6 and 12 months follow up; median PVR (ml) was 45 (20–77.5), 50 (20–90) and 45 (12–87.5) at 3, 6 and 12 months follow up. Median IPSS and IPSS QoL were 10 (6–13) and 2 (1–3), 10 (5.7–14) and 2 (1–3), 10 (5–16.5) and 2 (1–3) respectively at 3, 6 and 12 moths follow up.

From a sexual standpoint, antegrade ejaculation was preserved in all sexually active patients after the procedure, with an improvement in the median MSHQ-3 item score of 10 (5–13), 11 (5–14) and 9 (5–13) at each endpoint (*p* < 0.001), excluding patients with indwelling catheters.

**Fig. 1:**
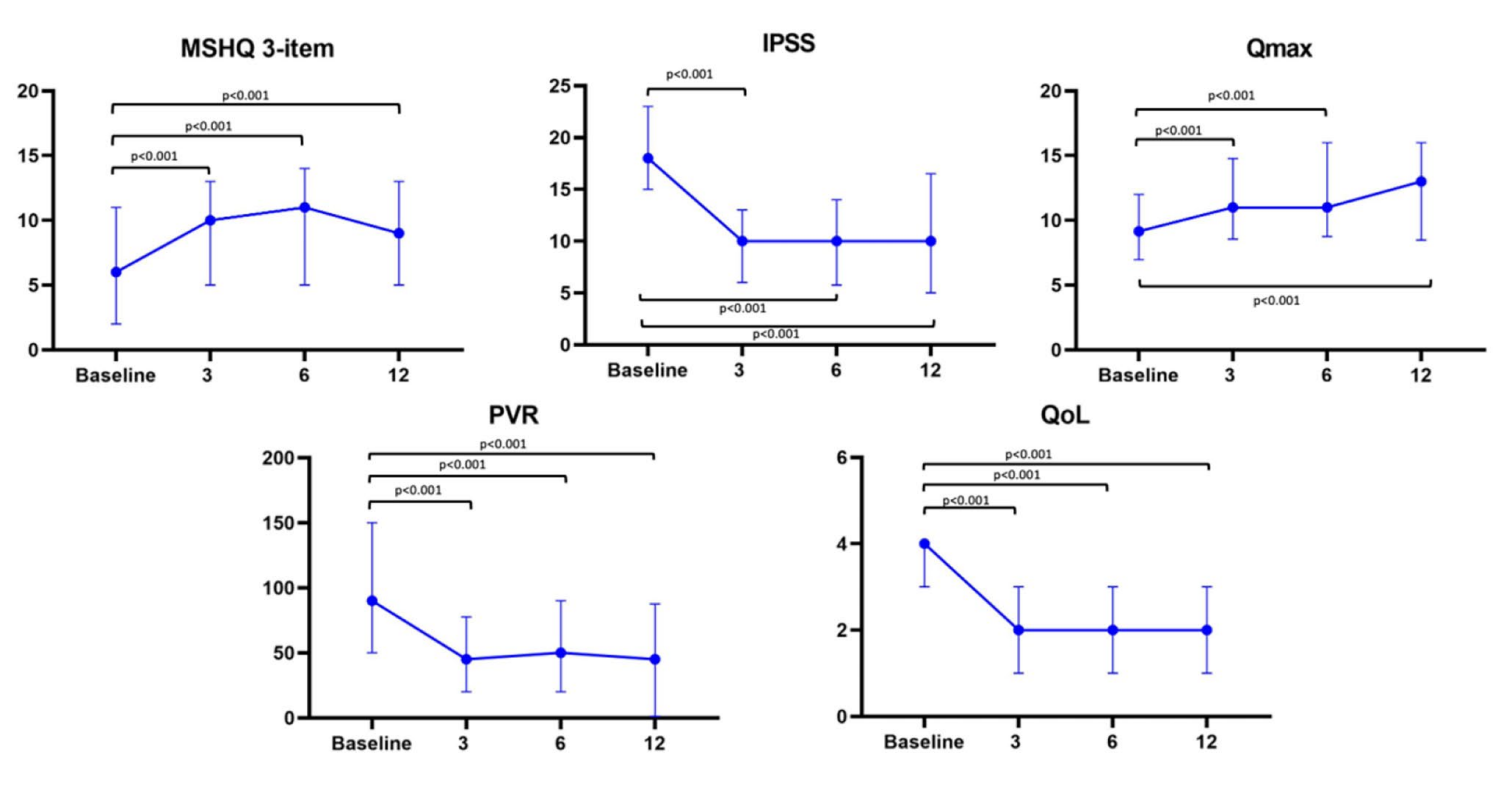
Clinical outcomes at 3, 6, 12 months. MSHQ male sexual healt questionnarie 3 items, IPSS International Prostate Symptoms Score, Qmax maximum urinary flow rate, PVR post-void residual, QoL quality of life

As illustrated by the Fig. 1, improvements remained consistent over time. Moreover, a clinically meaningful decrease in IPSS risk group (i.e. moderate to mild, severe to moderate, etc.) was shown at both the 3-mo and 12-mo time points (Supplementary Fig. 1).

Out of the 14 patients with indwelling catheter before TPLA, 5 (35.7%) continued to require permanent catheterization after the procedure. Of these, 3/5 succeded in catheter removal after shifting to other endoscopic surgery, while 2/5 maintained indwelling catheters (in one case for patient choice, in the other case for patient’s comorbidities) (Supplementary Table 1). The treatment failure rate at last follow up was 9%; in addition to the 2 cases who maintained indwelling catheters, in 7 cases, due to relapse of symptoms or non-negligible worsening of functional outcomes, an endoscopic procedure (TURP/Holep) was required. A detailed description of these patients is reported in Table 3.

**Table 3:** Overview of patients shifted to endoscopic surgery after TPLA

Progressive case number	Age (years)	ASA score	Comorbidities	Antiplatelet or anticoagulant therapy	Prostatic volume (ml)	Indwelling catheter before TPLA	Energy delivered (J)	Catheterization time (days)	Follow up (months)	Indwelling catheter after TPLA	Switch to other treatment	Indwelling catheter after other treatment
n. 25	65	1	none	no	80	yes	2100	-	18	yes	TURP	no
n. 26	73	1	none	no	50	no	2600	19	14	no	TURP	no
n. 50	62	2	Pacemaker carrier, sinus node dysfunction syndrome	no	90	no	2800	10	12	no	HoLEP	no
n. 60	54	2	Cardiac ablation for paroxysmal supraventricular tachycardia (PSVT)	no	50	no	2400	0	9	no	TUIP	no
n. 61	64	1	Hypertension	no	68	no	2400	3	9	no	TURP	no
n. 63	56	1	none	no	30	yes	2300	-	4	yes	TURP	no
n. 65	66	1	none	no	70	yes	3200	-	6	yes	TURP	no

## Discussion

The introduction of TPLA as a treatment for LUTS due to BPH represents a recent, minimally invasive solution, allowing to avoid the transurethral approach, reducing postoperative side effects and the potential for unfavorable outcomes [15]. Furthermore, has been shown to be feasible under local anesthesia alone and in an outpatient setting, not requiring hospitalization [13].

To date, TPLA has garnered an increasing body of research and consensus, although one of the main limitations of the studies in this clinical scenario is represented by the low number of patients enrolled. As such, also considering the relative lack of comparative trials, TPLA is not currently considered among the uMISTs by the most recent EAU Guidelines [3].

Recently, Laganà et al. [16] published the 12-month results of their case series, which included 63 enrolled patients, demonstrating a statistically significant improvement in IPSS, PVR, and Qmax at 12 months, in line with the 12-month results reported by other groups [17–19].

In the most recent single-center, prospective, randomized, open-label trial published by Bertolo et al., the 12-month results of 25 patients with similar characteristics undergoing TURP and 25 undergoing TPLA were compared. Once again, an improvement in uroflowmetry parameters was observed in both groups with a worsening of ejaculatory function in the TURP group compared to TPLA [20].

In this scenario, to the best of our knowledge, our study represents the largest prospective series of patients treated with TPLA, and highlights several key findings.

First, our study confirms the effectiveness of the procedure in terms of Qmax, PVR, and IPSS at a mid-term follow-up as previously described, and the overall minimal rate of complications [21]. Notably, while all patients underwent a standardized preoperative work-up to exclude those who needed/preferred more invasive treatment options, in this series we included a real-life patient population which may be reflective of contemporary clinical practice.

In this regard, our study included a highly heterogeneous cohort in terms of patient- and prostate-related factors, ranging from young patients seeking an alternative to pharmacological therapy in order to preserve ejaculation, to elderly and highly comorbid patients with a non-negligible perioperative risk. Therefore, this study highlights the feasibility, safety and efficacy of TPLA in different clinical scenarios with heterogenous clinical aims, allowing to tailor the management in light of the individual patient needs following the modern principles of personalized medicine.

Second, the results of TPLA in our series are at least non-inferior to previous evidence reporting the outcomes of other uMISTs such as Rezum [22, 23] and Aquabeam [24, 25].

Moreover, it could represent the initial step for the management of LUTS due to BPH without precluding potential future more invasive procedures (TURP/HoLEP); in this regard, in our series, for patients requiring other surgery after TPLA, urinary outcomes were not affected from the previous TPLA (Table 3).

Additionally, TPLA could be offered to elderly, highly comorbid patients who can benefit from an outpatient setting, avoiding hospitalization, general or spinal anesthesia, and not requiring suspension of “lifesaving” drugs like anticoagulants or antiplatelets [26].

Third, our study included 14 catheter-carrier patients, 9 of which achieved spontaneous micturition after the procedure, resulting in a success rate of 64% (Supplementary Table 1). Similar results were reported for other minimally invasive techniques such as PAE (65.4–73.1%) [27, 28], and slightly lower than the ones described after REZUM in several reviews (83%; 70.3–100%) [29, 30].

Finally, a potential advantage of this technique could rely in its cost-effectiveness (as compared to current gold standards), especially in public healthcare systems. In fact, even if the aim of this study was not to conduct a formal cost-effectiveness analysis, implementation of TPLA may result in reducing costs for BPH surgical management at least in a proportion of patients, with substantial benefits for waiting lists and costs of care.

In this regard, at our Centre, TPLA could save 1550 €/procedure as compared to TURP and 2600 €/procedure as compared to Holep (Supplementary Table 2).

Despite their novelty, our findings need to be interpreted with caution. In fact, several caveats and limitations could have influenced the study results. First and foremost, our real-life study cohort was carefully selected, yet heterogenous in patients’ and prostates’ characteristics; this may have introduced biases in the interpretation of the results. Secondly, the median 12-month follow-up is not a sufficient time frame to robustly confirm the solidity of the results; therefore, a continuation of the follow-up and additional analyses will be necessary. Finally, our findings could not be reproducible in other Centres and/or healthcare contexts, even considering the standardization of TPLA technique in our Institution during the learning curve [13, 15].

Acknowledged these limitations, our experience supports TPLA as an appealing technique for well-selected patients with LUTS due to BPO. In particular, being performed in an outpatient setting under local anesthesia, avoiding the need for an urethral access, it has the potential to minimize morbidity while ensuring favorable functional outcomes.

While a multicentre prospective registry on TPLA will be launched soon, further trials are needed to assess the comparative effectiveness of TPLA and other uMISTs and/or conventional endoscopic techniques for patients with LUTS due to BPO.

## Conclusions

TPLA represents a safe option for selected well-informed patients with LUTS due to BPO. Our prospective study confirms the feasibility and favorable perioperative and functional outcomes in a real-world cohort with heterogenous prostate volumes and patient characteristics. Larger multicenter studies will be necessary to define the role of TPLA in the contemporary algorithm for patients requiring treatment for LUTS due to BPO.

## Electronic supplementary material

Below is the link to the electronic supplementary material.

Supplementary Material 1

Supplementary Material 2

Supplementary Material 3: Fig. 1.— Study cohort’s change score of symptoms based on IPSS scores into mild (0–7), moderate (8–19), and severe (20–35) classes before and after surgery.

Supplementary Material 4
