# Supplementary material for: Transperineal laser ablation (TPLA) of the prostate for benign prostatic obstruction: the first 100 patients cohort of a prospective, single-center study
Source: World J Urol. 2024 Jul 10;42(1):402. doi: 10.1007/s00345-024-05077-z (PMC11236842; doi:10.1007/s00345-024-05077-z)
Supplement: Supplementary file 1 — Supplementary Material 1 [file 345_2024_5077_MOESM1_ESM.docx]

Supplementary Table 1.⎯ Characteristics and outcomes of catheter-carrier patients

| Progressive case number | Age | ASA score | Comorbidities | antiplatelet/anticoagulant therapy | Prostatic volume (ml) | Energy delivered (J) | Catheterization time (days) | Follow up (months) | Indwelling catheter after TPLA | Therapy at last followup | Switch to other treatment | Indwelling catheter after other treatment |
| --- | --- | --- | --- | --- | --- | --- | --- | --- | --- | --- | --- | --- |
| n. 11 | 81 | 3 | ischemic stroke | cardioaspirine | 120 | 2800 | 8 | 19 | no | Alpha blocker | No |  |
| n. 12 | 84 | 3 | bladder cancer, chronic kidney disease, solitary kidney, type 2 diabetes mellitus, glaucoma | no | 30 | 3000 | 95 | 19 | no | Alpha blocker + 5-ARI | No |  |
| n. 13 | 74 | 1 | none | no | 70 | 3000 | 10 | 20 | no | Alpha blocker | No |  |
| n. 18 | 86 | 2 | urotelial bladder cancer, type 2 diabetes mellitus | no | 40 | 2800 | 10 | 18 | no | no | No |  |
| n. 25 | 65 | 1 | none | no | 80 | 2100 | - | 18 | yes | no | TURP | no |
| n. 39 | 85 | 2 | hypertension, type 2 diabetes mellitus | cardioaspirine | 60 | 6400 | 10 | 16 | no | 5-ARI | No |  |
| n. 41 | 68 | 1 | none | no | 40 | 3000 | 30 | 15 | no | Alpha blocker | No |  |
| n. 48 | 61 | 1 | none | no | 65 | 3000 | 9 | 11 | no | no | No |  |
| n. 63 | 56 | 1 | none | no | 30 | 2300 | - | 4 | yes | no | TURP | no |
| n. 65 | 66 | 1 | none | no | 70 | 3200 | - | 6 | yes | no | TURP | no |
| n. 73 | 59 | 2 | moderate chronic kidney disease | no | 50 | 2200 | - | 4 | no | no | No |  |
| n. 75 | 93 | 3 | TIA | cardioaspirine | 39 | 2800 | 21 | 3 | no | Alpha blocker + 5-ARI | No |  |
| n. 92 | 82 | 2 | type 2 diabetes mellitus | no | 68 | 3200 | - | 4 | yes | no | No |  |
| n. 93 | 80 | 3 | heart failure, Parkinson's disease | NOAC | 40 | 3200 | - | 4 | yes | no | No |  |
