# Supplementary material for: Transperineal laser ablation (TPLA) of the prostate for benign prostatic obstruction: the first 100 patients cohort of a prospective, single-center study
Source: World J Urol. 2024 Jul 10;42(1):402. doi: 10.1007/s00345-024-05077-z (PMC11236842; doi:10.1007/s00345-024-05077-z)
Supplement: Supplementary file 2 — Supplementary Material 2 [file 345_2024_5077_MOESM2_ESM.docx]

Supplementary table 2: summary of costs for TPLA, HoLEP and TURP procedures at our centre. This cost assessment does not include fees for medical equipment, sterilizing process costs and eventual additional costs for intensive care unit postoperative management.

|  | **TPLA**   - **Ambulatory setting** - **60 min procedure** - **2 fiber used** - **Preoperative examination: routine bloody exams** - **No hospitalization needed** | **HoLEP**   - **Operative room setting** - **90 – 120 min procedure** - **1 holmium laser fiber used** - **Preoperative examination: Anesthesiology visit, routine bloody exams, ECG, chest RX** - **3 days hospitalization** | **TURP**   - **Operative room setting** - **60 – 90 min procedure** - **1 bipolar loops** - **Preoperative examination:** **Anesthesiology visit, routine bloody exams, ECG, chest RX** - **3 days hospitalization** |
| --- | --- | --- | --- |
| Histological examination cost | 0,00 | 150,00 | 150,00 |
| Preoperative examination cost | 80,00 | 150,00 | 150,00 |
| Operating room cost | 120,00 | 2700,00 | 1900,00 |
| Surgical equipment costs (diode laser fibers, holmium laser fibers, bipolar loops, etc.) | 700,00 (price per fiber) | 2000,00  (re-usable up to 10 times) | 500,00 |
| Hospitalization cost (considering standard practices at our Institution) | 0,00 | 1000,00 | 1000,00 |
| Total (euro) | 1600* | 4200** | 3250,00 |

*The total amount is calculated considering the use of 2 diode-laser fibers per procedure.

**The total amount is calculated considering the price of 1 holmium-laser fibre ideally re-usable up to 10 times for a single use.
