# Supplementary material for: Transperineal laser ablation (TPLA) of the prostate for benign prostatic obstruction: the first 100 patients cohort of a prospective, single-center study
Source: World J Urol. 2024 Jul 10;42(1):402. doi: 10.1007/s00345-024-05077-z (PMC11236842; doi:10.1007/s00345-024-05077-z)
Supplement: Supplementary file 3 — Supplementary Material 3: Fig. 1.— Study cohort’s change score of symptoms based on IPSS scores into mild (0–7), moderate (8–19), and severe (20–35) classes before and after surgery. [file 345_2024_5077_MOESM3_ESM.pdf]

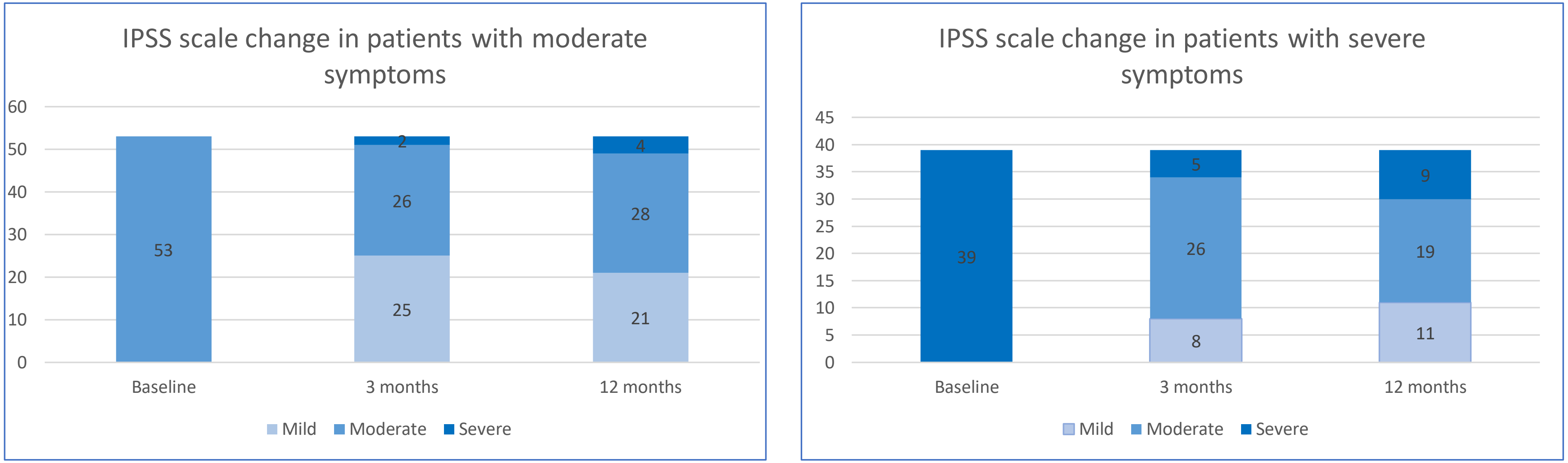

Supplementary Figure 1. Study cohort's change score of symptoms based on IPSS scores into mild (0-7), moderate (8-19), and severe (20-35) classes before and after surgery.
