# Supplementary material for: Transperineal laser ablation (TPLA) of the prostate for benign prostatic obstruction: the first 100 patients cohort of a prospective, single-center study
Source: World J Urol. 2024 Jul 10;42(1):402. doi: 10.1007/s00345-024-05077-z (PMC11236842; doi:10.1007/s00345-024-05077-z)
Supplement: Supplementary file 4 — Supplementary Material 4 [file 345_2024_5077_MOESM4_ESM.docx]

Reviewer #1: This is a case series describing results of transperineal laser ablation (TPLA) in 100 patients with BPH. There have been many gimmick surgeries for BPH similar to this one such as TUMT, TUNA, HIFU, urolift and rezum. At best these are considered "half-measure" procedures with efficacy somewhere between medication and TURP. There are several issues that should be addressed prior to acceptance of the manuscript for publication:

1. Follow-up duration is short at 12m. This is a major caveat as most data pertaining to previously failed treatments demonstrated similar short-term trends. This limitation needs to be clearly stated.

Thank you for the comment; the duration of the follow-up is certainly a limitation, and we therefore make it a point to emphasize it in the discussion. It is evident that these are preliminary data regarding this technique, and longer-term studies are needed to confirm or refute its effectiveness. Given the novelty of the procedure itself, there is currently no larger cohort with such a high median follow-up. For this reason, we find the discussion and publication of these data interesting and stimulating.

2. In the introduction it is stated that TURP is the gold standard for prostates up to 80ml and enucleation is the best option for patients with larger prostates. A reference to a trial published in BJU is cited to substantiate this. I don't think many urologists would agree with that statement. Enuncleations are rarely performed at most US centers and TURP or staged TURP is the mainstay for large glands. I do not think the cited reference reflects real world practice.

At the time of writing this article, in the EAU and AUA guidelines, it is clearly stated that the first-line treatment for prostates larger than 80 ml is enucleation, whether performed using endoscopic, open, laparoscopic, or robotic techniques.

<https://uroweb.org/guidelines/management-of-non-neurogenic-male-luts>

https://www.auanet.org/guidelines-and-quality/guidelines/benign-prostatic-hyperplasia-(bph)-guideline#x8208

However, we acknowledge that this may not be the global standard in real-world experience, and therefore, we have modified the relevant paragraph to clarify this point.

3. All data are expressed as medians and IQRs, and all p values are <0.001. This despite the values being very close together, for example 3 and 6m change in Qmax 2.4 (01-4.4) and 2.5 (0.8-5.9). This analysis method implies a difference when one probably does not actually exist. For continuous data means t-testing and ANOVA should be used.

In the mentioned case, as well as in the other values in the table, the comparison is always between the value at the specified time point and the baseline. Therefore, the p-value is not referring to the difference between 3 and 6 months change in Qmax but rather to 3 and baseline, and 6 and baseline, for example. Due to the small sample, we always assume in these cases a not continuous distribution, that’s why datas have been expressed in median and IQRs. We used a non-parametric test to compare the values at 3,6, and 12 months, each with the baseline value.

4. Similarly, in Figure 1 there appear to be favorable trends, but error bars are large. No p values are provided. These graphs require additional statistical analysis.

Figure 1 is the graphical representation of Table 2 (where p values are reported). The aim of Figure 1 is to provide a graphical representation of the main findings to allow the readers to catch the “overview” of the study findings. As such, we opted for not reporting a lot of p values in the Figure, which might have complicated its interpretation.

5. There is concern for selection bias. The manuscript states TPLA was conducted on 100 consecutive patients. Does this mean that 100 patients were not offered medical Tx or TURP but were relegated immediately to PTLA, or does it mean that men opted for PTLA and the study accounts for 100 cases?

Thank you for the comment; we will rephrase the statement more accurately. The phrase that TPLA was conducted on 100 consecutive patients implies that these 100 patients were treated sequentially in chronological order, presenting the data for each patient treated within the reported time frame. We clarified the potential for selection bias in the discussion.

Reviewer #2: I commend the authors for leading this project on a new uMIST for BPH.

INTRODUCTION:
- Correctly addresses the topic and the aim of the study.

Thank you for the comment; we will proceed to modify the final part of the introduction by specifying as clearly as possible the study's aim.

MATERIAL AND METHODS:
- Being the authors' center equipped with other uMIST techniques, why did the authors decide to perform a non-comparative study with other treatments?

Thank you for the comment; this is indeed the main point of reflection and the direction future research should take. In our series, we could not design a comparative study with other uMISTs (i.e. UroLift, Rezum, etc.), as they are currently not available at our Hospital. The comparison with other uMIST techniques is already the subject of a multicentric study that has been developed by the Italian Society of Urology, which is actively recruiting.

- Within the inclusion criteria the authors should report how the prostate volume threshold was established.

Thank you for the clarification; we have modified the text accordingly. There was a mistake in the upper limit of the prostate volume allowed for this study, which is 100 ml and not 160 ml. We thank the Reviewer very much for allowing us to revise this section. About the threshold itself, up to date not a real consensus neither from a Delphi consensus neither from the company. We empirically determined the cut-off based on indications from other MISTs (such as Urolift, as mentioned, and Rezum). Inizio modulo

Fine modulo

- Can the authors briefly describe their technique with this new treatment which is not adopted in many centers? Despite citing previous experience, this should be explained to readers not confident with this approach.

We have added a brief description of the methodology. Due to length constraints imposed by the journal, we cannot provide a detailed description of the technique in this text. Therefore, we are compelled to refer to the comprehensive description provided in previous articles.

- I won't use the treatment-free rate as an outcome measure, but treatment failure. Indeed, some patients required a catheter again after treatment.

We thank the Reviewer very much for this comment. We have modified the term “treatment-free rate” with “treatment-failure”, which is a clearer outcome measure for readers.

- After how many procedures the surgeon was proficient with the technique?

Great observation. In our case series, the operators are all professionals with advanced experience in transperineal fusion biopsy, each having performed a minimum of 100 procedures. In this setting, the learning curve to conduct an efficient procedure has been estimated to be around 5 procedures, although a dedicated analysis has not been conducted due to the following reasons: 1) presence of multiple surgeons; 2) lack of evidence on how to structure the steps of the learning curve to identify the performance metrics. However, we are grateful to the Reviewer for his/her suggestion, which prompted us to design a study in this regard.

- The Mann-Whitney test is used for the comparison of two continuous variables with non-parametric distribution, but I see only comparisons of three groups. Did the authors first compare the baseline with each time point, and after compare all the groups? Please better specify which test and in what case each test was adopted.

Thank you for the insightful comment. We have clarified this relevant point in the manuscript. We used a non-parametric test to compare the values at 3,6, and 12 months, each with the baseline value. We did not compared the values at 3, 6 and 12 months to each other.

RESULTS:
- Looking at costs I see a hospitalization time for HoLEP which is 3 days, increasing the costs. Why such a long hospital period for a procedure that allows the patient discharged on the first post-operative day and in some countries the same day? The authors should at least discuss this in the limitations.

This comment is indeed very relevant. As specified in the text, the study was not designed to provide a formal cost-effectiveness analysis. The comment on the LOH after Holep is very pertinent. Yet, in our Centre (and in general in our healthcare system), the usual LOH after Holep is indeed 3 days, meaning that the catheter is removed on POD 3 and the patient is discharged without the catheter on the same day. We acknowledge that this practice might be different in other Centres/Countries, but our aim was to provide an idea of how TPLA could be compared to Holep in our Centre. Assessing the heterogeneity of LOH across Centres/Countries after uMISTs and Holep is definitely a great topic for future studies.

DISCUSSION:
- Compared to other uMIST, can the authors provide what should lead a physician to prefer this treatment over the others? The outpatient setting? The lower costs? The lower invasiveness? The authors should discuss about this.

Thank you for the comment; we have included a brief description of the advantages of this technique compared to other proposed minimally invasive techniques in the discussion. Overall, we believe the main advantage of TPLA is to be an outpatient procedure, under local anesthesia, with no need for urethral access, and potentially lower costs as compared to other uMISTs.
